# Multi-Disciplinary Management in Rectal Cancer Survivorship: A Clinical Practice Review

**DOI:** 10.1007/s12029-022-00885-1

**Published:** 2023-01-09

**Authors:** Hilary Chan, Marissa B. Savoie, Amir Munir, Javid Moslehi, Mekhail Anwar, Angela Laffan, Tami Rowen, Rebeca Salmon, Madhulika Varma, Katherine Van Loon

**Affiliations:** 1 Department of Medicine, Division of Hematology and Oncology, University of California, San Francisco (UCSF), 550 16th Street, Floor 06, Room 6803, Box 3211, San Francisco, CA 94158 USA; 2 UCSF Helen Diller Family Comprehensive Cancer Center, San Francisco, CA USA; 3 Department of Medicine, Massachusetts General Hospital, Boston, MA USA; 4 Department of Medicine, Division of Cardiology, UCSF, San Francisco, CA USA; 5 Department of Radiation Oncology, UCSF, San Francisco, CA USA; 6 Department of Electrical Engineering and Computer Sciences, University of California, Berkeley, Berkeley, CA USA; 7 Department of Obstetrics and Gynecology, UCSF, San Francisco, CA USA; 8 Section of Colorectal Surgery, Department of Surgery, Department of Medicine, UCSF, San Francisco, CA USA

**Keywords:** Rectal cancer, Survivorship, Toxicity, Quality of life, Modifiable risk factors

## Abstract

Colorectal cancer (CRC) is the third most common cancer in the USA and worldwide. In the USA, nearly one-third of CRC cases are anatomically classified as rectal cancer. Over the past few decades, continued refinement of multimodality treatment and the introduction of new therapeutic agents have enhanced curative treatment rates and quality of life outcomes. As treatments improve and the incidence of young onset rectal cancer rises, the number of rectal cancer survivors grows each year. This trend highlights the growing importance of rectal cancer survivorship. Multimodality therapy with systemic chemotherapy, chemoradiation, and surgery can result in chronic toxicities in multiple organ systems, requiring a multi-disciplinary care model with services ranging from appropriate cancer surveillance to management of long-term toxicities and optimization of modifiable risk factors. Here, we review the evidence on these long-term toxicities and provide management considerations from consensus guidelines. Specific topics include bowel dysfunction from radiation and surgery, oxaliplatin-induced neuropathy, accelerated bone degeneration, the impact of fluoropyrimidines on long-term cardiovascular health, urinary incontinence, sexual dysfunction, and psychosocial distress. Additionally, we review modifiable risk factors to inform providers and rectal cancer survivors of various lifestyle and behavioral changes that can be made to improve their long-term health outcomes.

## Introduction

In 2021, an estimated 45,230 cases of rectal cancer were diagnosed in the USA [1]. Of these, approximately two-thirds of cases are diagnosed at a localized or locoregional stage and are amenable to treatment with curative intent [1]. Five-year survival rates for localized and locoregional disease are currently 90.1% and 73.8%, respectively [1]. Among colorectal cancer (CRC) survivors in the USA, nearly one-third are rectal cancer survivors [2]; however, this proportion varies worldwide due to geographic variation. In addition, while the overall incidence of rectal cancer has decreased in the USA over the past 20 years with the incorporation of screening into routine clinical practice, the incidence of young-onset rectal cancer, defined as rectal cancer diagnosed in patients less than 50 years of age, has risen over the past decade [3, 4].This striking trend reflects a shifting demographic in our survivor population and further highlights the importance of survivorship care moving forward.

Curative treatment for locoregional rectal cancer entails a multimodality approach with a combination of surgery, chemoradiation, and chemotherapy [1, 5]. Growing evidence supports the use of total neoadjuvant therapy (TNT), which involves pre-operative systemic chemotherapy with capecitabine/oxaliplatin (CAPOX) or 5-fluorouracil (5-FU)/leucovorin/oxaliplatin (FOLFOX) and chemoradiation with oral capecitabine or infusional 5-FU prior to curative-intent resection. TNT is associated with improved chemotherapy tolerance and completion as well as higher pathologic complete response rates, allowing for less extensive surgeries and potential preservation of rectal function [5–7].

**Fig. 1:**
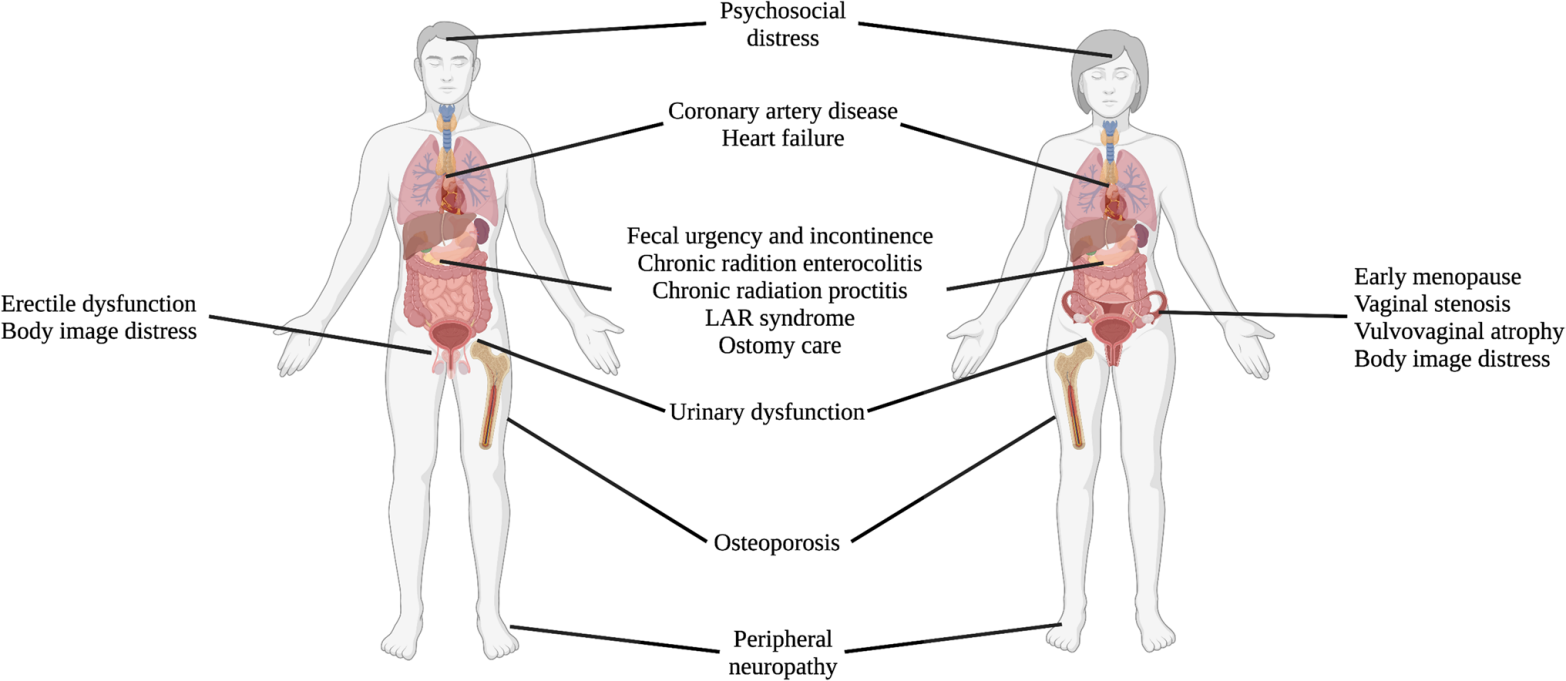
Summary of organ systems affected by long-term toxicity in rectal cancer survivorship

As the number of rectal cancer survivors continues to rise, attention to survivorship and its impact on quality of life (QOL) is increasing. Data from the National Surgical Adjuvant Breast and Bowel Project R-04 trial demonstrated that physical and mental well-being scores significantly worsened from baseline after neoadjuvant chemoradiation and that these changes may persist up to 5 years post-treatment in subsets of rectal cancer survivors [8]; thus, this is a high priority area in survivorship care. Herein, we provide a comprehensive evidence-based review characterizing and describing aspects of rectal cancer survivorship care by organ system (see Fig. 1) and provide readers with strategies for management of chronic toxicities.

## Chronic Toxicities by Organ System

### Gastrointestinal Toxicity

#### Complications Related to Radiotherapy

Chronic gastrointestinal toxicity from radiotherapy (RT) typically presents between 3 months and 1 year after RT and can persist for years and even decades [9]. Patients who receive pre- or post-operative RT compared to those who underwent surgery alone have significantly higher rates of diarrhea and fecal incontinence [10]. The mechanism of injury in acute radiation gastropathies is thought to be mediated by inflammation, while RT-induced chronic injury to the small and large bowel is often mediated by small vessel ischemia and fibrosis, leading to altered absorption and motility [11].

A recent systematic review characterized the most common late RT-induced adverse events as diarrhea (up to 35%), fecal incontinence (22%), rectal bleeding (9%), rectal pain (13%), and bowel obstruction (7.4%) [12]. While the shift from post-operative to pre-operative RT and advances in radiation delivery techniques have reduced rates of locoregional recurrence and lowered rates of late gastrointestinal toxicity [12–14], QOL remains impacted in rectal cancer survivors [15, 16]. Below we discuss both chronic radiation enteritis and proctitis. While symptoms between the two entities may overlap, rectal cancer survivors are far less likely to have small bowel complications given that the location of radiation therapy typically spares most of the small intestine unless there is unfavorable anatomy or a bulky and/or proximal rectal tumor.

Chronic radiation enteritis may present with nausea, vomiting, abdominal pain, and diarrhea. Fistulas, abscess formation, and perforation represent a less common, though more severe presentation [11]. While rare in rectal cancer survivors given the location of radiotherapy, direct damage to enterocytes may induce bile-salt and carbohydrate malabsorption, while changes in intestinal motility have been implicated in the development of small intestinal bacterial overgrowth (SIBO) [17, 18]. Diagnosis of these entities involves carbohydrate breath testing as well as stool cultures [19]. Cross-sectional imaging can be pursued for non-specific symptoms of bloating, weight loss, and abdominal pain [11]. Medical treatment, depending on exact etiology, can range from dietary modification, bile acid sequestrants, antibiotics, and/or anti-diarrheals [17]. Referral to gastroenterology for endoscopic evaluation of persistent symptoms can be considered. Argon plasma coagulation during endoscopy can be used to address bleeding from telangiectasias associated with chronic radiation enteritis, while surgical interventions may be required for intestinal obstruction, strictures, fistulas, and refractory bleeding [11, 20].

Chronic radiation proctitis can present with diarrhea, tenesmus, rectal discharge, rectal bleeding, fecal urgency, and fecal incontinence. Endoscopic evaluation will reveal stigmata of radiation proctitis, including vascular ectasias, mucosal pallor, and tissue friability [21], but is also useful in ruling out infectious etiologies, recurrent or new primary malignancies, and new-onset inflammatory bowel disease, which occurs in 4% of patients in the post-RT setting [17]. Non-invasive treatments for chronic radiation proctitis include anti-inflammatory/steroid enemas, sucralfate enemas, oral metronidazole combined with colonic irrigation, and oral vitamin and fatty acid chain supplementation, while more invasive measures, like argon plasma coagulation, can be employed during endoscopy [22, 23]. Hyperbaric oxygen is recommended by the American Society of Colon and Rectal Surgeons and can be used in refractory cases [22].

In our practice, for a patient with suspected gastrointestinal radiation toxicity, we first evaluate for infection with stool studies. Once infection is excluded or symptoms are refractory despite adequate antibiotic treatment, we recommend diet modification with a low residue diet. Use of anti-diarrheals is also recommended if increased stool frequency is the predominant symptom. We prefer the use of sucralfate retention enemas (2 g of sucralfate suspension mixed with 50 ml of tap water) administered twice daily for radiation proctitis. We instruct patients to inject the enema into the rectum and then lie down for at least 5 minutes on each of the following sides: front, right, left, and back. We also provide additional instruction to lie on the front side for as long as possible as to allow the sucralfate to coat the front wall of the rectum, where a significant amount of RT-related toxicity occurs. If symptoms remain refractory, referral to gastroenterology for additional evaluation should be considered. A referral for hyperbaric oxygen for refractory symptoms can be pursued, when available.

#### Complications Related to Surgery

**Fig. 2:**
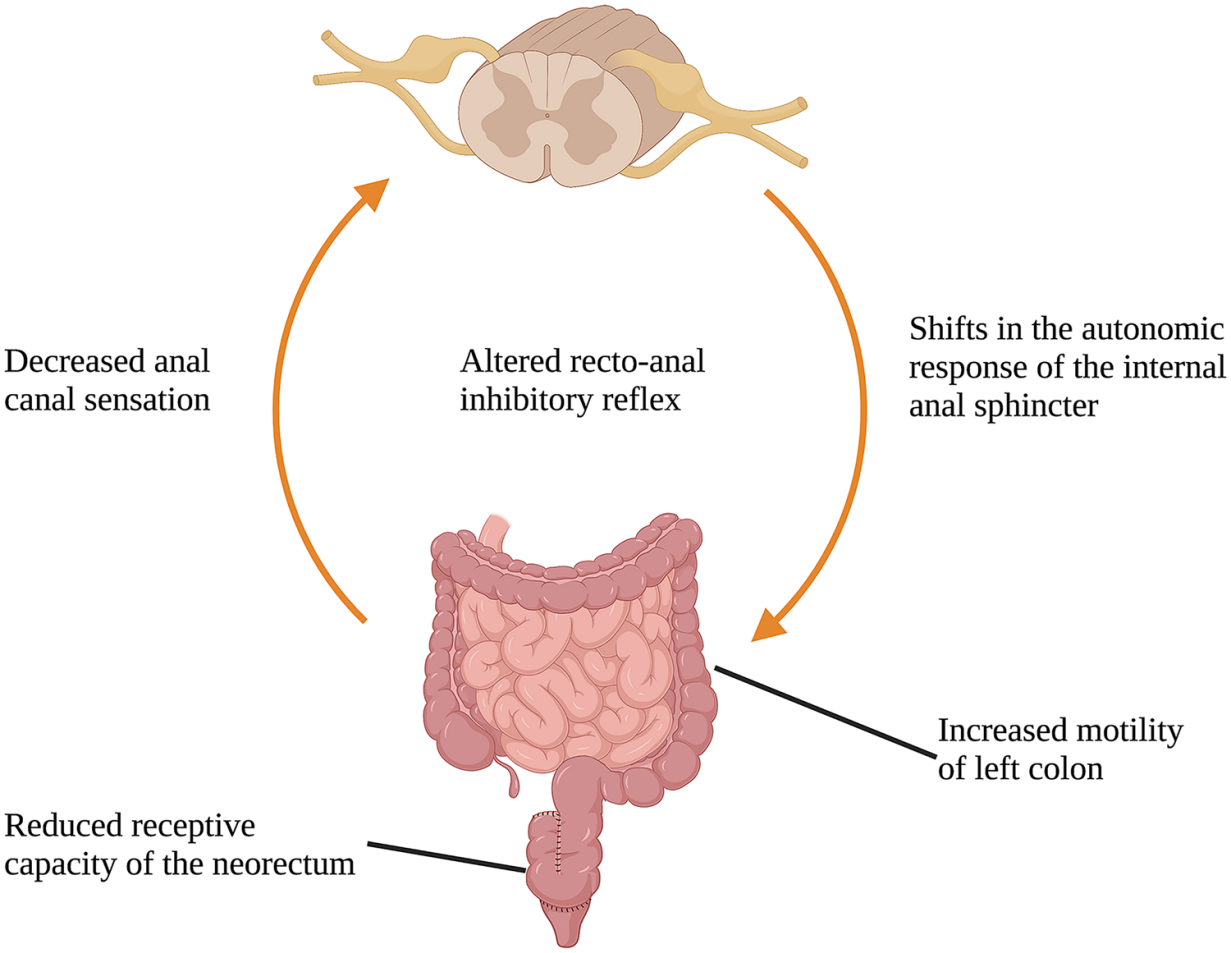
Various mechanisms in pelvic nerve alterations after surgery that lead to impaired bowel function

Patients with resectable rectal cancer typically undergo one of two surgeries: (1) abdominal perineal resection (APR), which involves removal of the rectum and sigmoid colon and creation of a permanent stoma, or (2) low anterior resection (LAR), which involves altered restoration of intestinal continuity. Total mesorectal excision (TME), a standard principle of colorectal surgery involving mid- to low rectal cancers, requires resection at the level of the pelvic floor and potentially further. As a result, alterations in anatomy and the innervation of the neorectum may impair its ability to manage stool. These motor and sensory changes are detailed in Fig. 2 and result in the constellation of symptoms that define low anterior resection syndrome (LARS) [24]. Symptoms of LARS include unpredictable bowel habits, variable stool formation, sensation of incomplete evacuation, and increased stool frequency with associated symptoms of clustering, urgency, and incontinence [24]. Symptoms vary widely among patients with an estimated 41–58% of patients reporting major LARS syndrome [25, 26]. Severity has been directly correlated with the height of anastomosis and prior exposure to radiation [27]. Given the high incidence of LARS and its impact on QOL, patients often require intensive clinical support as they recover.

Appropriate pre-operative and post-operative patient education and guidance on expectant symptoms are key to initial management of bowel dysregulation after surgery as these symptoms typically improve over time [26]. Management of LARS is built on understanding the anatomic and resultant physiological changes in bowel habits. Bulking agents can improve stool packaging and reduce stool frequency, while the use of anti-diarrheal medications can modulate hyper-motility of the left colon. Reduction of nighttime stooling can be achieved using anti-diarrheal medications and/or low-dose opioids. Serotonin 5-HT3 receptor antagonists have also shown improvements in stool clustering and frequency, while bile acid sequestrants, such as cholestyramine and colesevelam, can be used to bind stools to reduce stool frequency [27, 28]. Lastly, use of barrier creams and guidance on gentle cleansing can support skin care in the perineum, which can be inflamed due to increased bowel activity. These recommendations are summarized in Table 1.

**Table 1:** A stepwise approach to management of bowel dysfunction

**Intervention**	**Mechanism**	**Indication**
Fiber (soluble or psyllium)	Stool binding	Stool frequency
Bile acid sequestrants (cholestyramine, colesevelam)	Stool binding	Stool frequency
Anti-diarrheals (diphenoxylate/atropine, imodium)	Slow GI motility	Stool frequency and clustering
5-HT3 antagonists (ramosetron)	Slow GI motility	Stool frequency and clustering
Biofeedback therapy	Neuromodulation	Stool frequency and fecal incontinence
Acetaminophen with low dose codeine *(use sparingly)*	Slow GI motility	Refractory fecal incontinence, stool clustering, and frequency

For patients with significant LARS, pelvic floor therapy, biofeedback, and sacral neuromodulation can be explored to reduce stool frequency and fecal incontinence [27]. Transanal irrigation, which involves left-sided colonic irrigation to reduce time between bowel movements, can be performed by patients with more significant symptoms [29]. Referral to gastroenterology to investigate alternative etiologies for changes in bowel movements may be indicated in cases of persistent symptoms. In addition, referral to a dietician can be considered at any point to identify potential dietary changes that could promote normal bowel movements. Finally, conversion to a permanent stoma can be considered in severe LARS [27].

Ostomy management represents a major factor in the QOL of rectal cancer survivors, as discussed below in the section on psychosocial well-being. Ostomy irrigation, a technique during which patients self-administer a water enema through their stoma to stimulate colonic peristalsis and thus a bowel movement, may provide patients an extended time without fecal discharge through the stoma and has been shown improve QOL in patients with permanent ostomies [30]. Consultation with an ostomy specialist should be obtained for additional guidance on ostomy irrigation as not all patients may derive benefit from this technique. For example, patients with end ileostomies may have persistent incontinence into their ostomy as they may not have enough large bowel intact to hold fecal matter. Ostomy support groups for rectal cancer survivors offer patients a shared community for emotional and practical guidance on ostomy management. In-person support groups are provided by local chapters of the United Ostomy Associations of America (UOAA), while virtual support groups and resources can be found on the UOAA website (https://www.ostomy.org/) [31]. Referrals to providers specializing in ostomy care (i.e., ostomy nurses) or colorectal surgeons can help address potential complications related to ostomy placement and management.

### Neurologic Toxicity

Oxaliplatin neurotoxicity is a cumulative toxicity that typically develops during later cycles of chemotherapy and can persist months and even years afterwards. These symptoms typically increase in severity with cycle 2 and persist throughout their treatment course with oxaliplatin, which is known to have a cumulative dose-dependent and dose-limiting side effect [32]. Dose-limiting toxicity typically occurs after cumulative oxaliplatin doses between 667 and 810 mg/m^2^, which corresponds to eight or more cycles of oxaliplatin [33–35]. Given that rectal cancer survivors undergoing TNT generally receive up to eight cycles of oxaliplatin, only a portion of rectal cancer survivors experience this long-term toxicity.

Chronic oxaliplatin-induced neuropathy is described as long lasting and unlikely to be reversible [36]. It is more likely to occur in those who experienced these symptoms in the acute setting [32]. It has been described to progress up to 3 months after oxaliplatin discontinuation with slow recovery of injured peripheral nerves afterwards. While numbness and tingling typically occur in the hands more than feet during active treatment, chronic symptoms more commonly persist in the feet, representing an anatomic reversal upon cessation of oxaliplatin [32].

According to the American Society of Clinical Oncology (ASCO) Practice Guidelines for Chemotherapy Induced Peripheral Neuropathy (CIPN), there are no evidence-based medical interventions for prevention and treatment of CIPN [37]. Thus, we recommend careful monitoring of this symptom during treatment and early dose reductions of oxaliplatin at the point at which symptoms are persistent and no longer cold-induced, while also balancing the potential benefits of treatment in the case of curable disease.

In patients with painful CIPN, duloxetine, a selective serotonin-norepinephrine re-uptake inhibitor (SNRI), is the only intervention with randomized controlled trial data demonstrating efficacy. CALGB 170601 was a phase 3 cooperative study in which patients with painful CIPN were randomized to duloxetine vs. placebo. A significant reduction in pain from oxaliplatin-induced peripheral neuropathy was detected, with an effect size of 0.5 [38]. High emotional function was shown to be a predictor of response to duloxetine in this study [39]. For our patients who develop painful CIPN, we typically recommend duloxetine 30 mg daily for 1 week and then increase the dose to 60 mg daily afterwards for CIPN as was done in the CALGB 170601 study [38].

Other therapies have been studied, though with low quality and without RCT level evidence; these include exercise therapy, acupuncture, scrambler therapy, topical neuro-acting agents, and oral cannabinoids [37]. The treatment landscape for oxaliplatin-induced neuropathy is relatively lacking, and we strongly recommend attention to the development of this symptom during chemotherapy administration. Further studies are needed to improve management of this debilitating side effect in rectal cancer survivors.

### Accelerated Bone Degeneration

Radiation therapy has been associated sacral and pelvic insufficiency fractures (PIF) in rectal cancer survivors [40]. While this topic is best studied in those with gynecologic and prostatic malignancies, patients undergoing pelvic RT for rectal cancer have an increased risk of PIF (HR 1.25) compared to those treated without RT [40]. Proposed mechanisms of action include decreased osteoblastic activity, increased osteoclastic activity, and damage to the bony matrix [41].

Often viewed as a late complication of radiation, PIF typically occur in the first 2 years after treatment, though can occur as early as a few months to as late as 8 years after completion of RT [41]. Reported incidence rates of radiation-induced PIF range from 3 to 34% in rectal cancer survivors [42]. Female sex, advanced age, and post-menopausal status are additional risk factors for developing PIF related to radiotherapy with a near doubling of PIF incidence among women in some studies [40, 43, 44]. It has been postulated that chemotherapy, often given in isolation or with radiotherapy, may trigger menopause in peri-menopausal woman, which exacerbates the risk of developing osteoporosis and osteoporotic fractures.

ASCO Clinical Guidelines for Management of Osteoporosis in Nonmetastatic Cancer Survivors recommend dual-energy X-ray absorptiometry (DEXA) screening to evaluate bone mineral density in patients who have traditional pre-existing risk factors for osteoporosis, recognizing that cancer treatment, such as radiotherapy and chemotherapy exposure, may put patients at further risk for developing osteoporosis and suffering insufficiency fractures [45, 46]. Non-pharmacologic interventions such as adequate calcium and vitamin D supplementation, physical activity with attention to weight bearing exercises, and alcohol and tobacco cessation represent potential alterations to modifiable risk factors that can be used to reduce the risk of PIF in rectal cancer survivors. Bone health agents, such as bisphosphonates and denosumab, may also be used in patients with osteoporosis to reduce the risk of fracture, as indicated [45].

### Cardiovascular Toxicity

Fluoropyrimidines, including 5-FU and capecitabine, are associated with acute coronary vasospasm. More recent data suggest that CRC survivors may be at some increased long-term risk of cardiovascular disease (CVD). A retrospective cohort study utilizing the SEER-Medicaid database showed that a 10-year cumulative incidence of new onset congestive heart failure (CHF) was 54.5% in older CRC survivors (age > 65 years) compared 18% in matched patients without a history of cancer. In addition, this study found that hypertension (HR 1.11), diabetes (HR 1.22), and exposure to radiation (HR 1.18) in CRC survivors were associated with an increased risk of new onset CVD [47]. Interestingly, this study also found that in older colorectal cancer survivors (age > 65 y), capecitabine alone was associated with an increased risk of CHF (HR 1.57), but a decreased risk of CVD (HR 0.72) compared to those receiving 5-FU alone at 2 years after their initial diagnosis [47]. Currently, there is a lack of prospective data investigating the risk of CVD in survivors who have received fluoropyrimidines as part of their chemotherapy regimen, and it is unknown if this increased risk for CVD also exists for younger CRC survivors.

Nonetheless, given the overlapping risk factors of age, hypertension, and diabetes, which predispose patients to both cardiovascular disease and cancer, the crux of CVD management for rectal cancer survivors is aggressive risk reduction through primary prevention [48]. Myocardial infarction, while rare, can occur with 5-FU coronary vasospasm and result in long-term cardiomyopathy. Rectal cancer survivors with concomitant heart failure with reduced ejection fracture should undergo guideline-directed medical therapy as per the American College of Cardiology and American Heart Association Guidelines [49].

The National Comprehensive Cancer Network (NCCN) guidelines on Cancer Survivorship recommend an “ABCDE” approach. This includes **A**wareness of the risk factors and symptoms of heart disease, **A**ssessment of being at risk for or having heart disease, **A**spirin use as needed; Blood pressure management; **C**holesterol management, **C**igarette and tobacco cessation, **D**iet and weight management, **D**iabetes prevention and treatment; **E**xercise**, E**chocardiogram, and **E**lectrocardiogram as needed [50]. As CRC and CVD share modifiable risk factors such as tobacco use, obesity, and sedentary lifestyle, active risk reduction is paramount to rectal cancer survivorship. Furthermore, screening for coronary artery disease, peripheral artery disease, and heart failure with a careful history and physical exam is essential. Both lifestyle modification and pharmacological treatments, if necessary, are recommended for aggressive primary risk reduction and represent a cornerstone of cardiovascular health in rectal cancer survivors.

### Urinary Dysfunction

Urinary dysfunction in rectal cancer survivors can present as abnormalities in bladder filling, voiding, and incontinence. Roughly one-third of rectal cancer survivors report urinary dysfunction, though some studies have reported symptoms of bladder dysfunction in nearly 60% of rectal cancer survivors with rates increasing 1 year after rectal cancer diagnosis [51, 52].

The mechanism of injury leading to urinary dysfunction is thought to be secondary to nerve damage during surgical resection of rectal tumors [53]. Studies have suggested worse urinary function in patients undergoing APR compared to those undergoing LAR; however, when controlled for other risk factors, these were not statistically significant [52, 54, 55]. The research on the impact of radiation on urinary dysfunction in rectal cancer survivors is mixed [51, 54, 56]. Menopausal changes associated with chemotherapy and radiation can also lead to urinary dysfunction in women [57, 58].

Several studies have explored risk factors associated with developing urinary dysfunction. Commonly identified risk factors include female sex, baseline urinary incontinence, physical inactivity, and the presence of other medical comorbidities [52, 54]. This is particularly pertinent for elderly patients, who have higher rates of urinary incontinence at baseline [59].

Urinary incontinence is typically characterized into three categories: urge, stress, and overflow incontinence. Urge incontinence may arise from damage to autonomic nerves resulting in detrusor muscle overactivity, while stress incontinence may be resultant from anatomic alterations after surgery [53, 60]. Overflow incontinence is generally less common, but can also arise from nerve damage during surgery [60]. Studies on management of urinary incontinence in rectal cancer survivors are generally lacking, so management is largely extrapolated from the general population.

Pelvic floor rehabilitation has been shown to improve urinary dysfunction in women and is generally recommended as a first line treatment in all types of urinary incontinence, particularly stress incontinence [61]. Pharmacologic treatment for urge incontinence typically involves anti-muscarinics, such as oxybutynin, or beta-3-adrenergic agonists, such as mirabegron, though caution is advised with both classes of medications in frail and elderly patients [53, 62]. Duloxetine has shown some benefit in stress incontinence and associated improvement in QOL, though long-term benefits remain unclear [63]. Evaluation by urology or urogynecology is also recommended as advanced therapies, such as sacral neuromodulation, botulinum injections, peri-urethral bulking injections, and reconstructive surgeries can be explored to treat refractory urinary incontinence [53]. For patients with recurrent urinary tract infections (UTI) related to atrophic vaginal tissue, vaginal estrogen cream has been shown to reduce the number of recurrent UTIs [64, 65]. In our practice, we recommend referral to pelvic floor rehabilitation for all rectal cancer survivors with bladder dysfunction. If symptoms persist, referral to urology or urogynecology is recommended for additional evaluation and potential pharmacologic and interventional treatment options.

### Sexual Dysfunction

Sexual dysfunction is common after rectal cancer diagnosis and can persist for years after treatment. The prevalence of sexual dysfunction is challenging to define due to varying outcome measures, differing norms around what constitutes dysfunction, and variable rates of pre-cancer sexual dysfunction. Sexual dysfunction is complex and can be affected by biological, psychological, and social factors, all of which can be affected by rectal cancer diagnosis and treatment.

Rates of sexual dysfunction after treatment for rectal cancer have been reported to be 66% in men and 42–60% in women [66, 67]. In an observational study of American patients treated for rectal cancer, pre-treatment rates of sexual activity were 70% among men and 64% among women, compared with 55% among men and 49% among women at 1 year after surgery; lower rates of sexual activity persisted at 5 years post-operatively [68]. It has been hypothesized that damage to pelvic autonomic nerves during tumor resection can lead to sexual dysfunction after rectal cancer treatment. However, several studies of patients with rectal cancer found lower sexual function before surgery compared to normative populations, and intra-participant difference in post-operative sexual functioning was not observed [69, 70]. Surgery and pelvic radiation can affect bowel habits, and fecal incontinence related to treatment has been associated with body image distress and decreased sexual functioning [67].

For female patients, chemotherapy and radiation suppress gonadal function, leading to decreased estradiol and testosterone production, and are associated genitourinary changes of menopause, which can cause dyspareunia or vulvar irritation. Younger age is generally associated with improved sexual functioning after treatment, and, rarely, women recover ovarian function after treatment [71]. Discomfort related to vulvovaginal atrophy is generally treated with topical lubricants or vaginal estrogen, which is safe and effective in patients with rectal cancer. Pelvic radiation can induce vaginal stenosis in which the vaginal canal loses elasticity, and often patients experience dyspareunia. Vaginal dilators for vaginal stenosis and sexual dysfunction after radiation are commonly offered at cancer center sexual health programs, although there is limited evidence of efficacy, which is most likely related to variability in compliance [72]. Early referral to gynecology is recommended in women who are receiving treatment for rectal cancer. Within 4 to 8 weeks of completing pelvic radiotherapy, we typically recommend starting vaginal estrogen and vaginal dilator use or resumed sexual activity to prevent vaginal stenosis and preserve sexual function. For patients with symptomatic vulvovaginal atrophy, we recommend topical vaginal estradiol 1 gram daily for 2 weeks, then application three times a week afterwards. Other formulations of estrogen such as vaginal estrogen tablets, administered similarly to topical estrogen, or vaginal estrogen containing rings, which are exchanged every 90 days, can be used as well.

For male patients, erectile function may be influenced by damage to pelvic autonomic nerves and/or microvasculature during resection or pelvic radiation. Ultimately, the treatment for cancer-associated sexual dysfunction in men is similar to standard treatment of erectile dysfunction which includes an assessment for psychological or situational precipitants and management of contributory risk factors such as diabetes mellitus, tobacco use, or hypertension. Phosphodiesterase-5 inhibitors have been shown to be effective in treating erectile dysfunction associated with brachytherapy or radiation for prostate cancer, which can be extrapolated to rectal cancer patients [73–75]. In our practice, treatment with sildenafil or tadalafil can be considered in patients without significant cardiovascular disease or concurrent use of contra-indicated medications (i.e., nitrates). We also recommend referral to urology for additional testing and evaluation for pharmacologic and advanced interventional techniques.

Sexual dysfunction is often multifactorial, and some have proposed the biopsychosocial model of sexual dysfunction to facilitate holistic treatment approaches. Body image distress related to treatment sequelae or presence of an ostomy may influence a patient’s approach to sexual activity [67, 76, 77]. Behavioral interventions such as supportive counseling around coping with sexual challenges have been evaluated in small studies, and improvements in sexual function were observed [78–80]. A summary of the sexual issues discussed is organized in Table 2.

**Table 2:** Management of sexual dysfunction in male and female patients

**Problem**	**Description**	**Management**
Early menopause	Most likely to occur in peri-menopausal patients undergoing chemotherapy	Younger female patients may have return of menses upon cessation of chemotherapy
Infertility	Damage to pelvic nerves during radiation. Impaired gamete production from chemotherapy	Sperm banking and oocyte retrieval should be explored in patients prior to receiving chemotherapy. Referral to reproductive specialists to evaluate infertility pre- and post-treatment should be considered
Vaginal stenosis	May be resultant of radiation therapy	Referral to gynecology for vaginal dilator therapy can be considered
Vulvovaginal atrophy	May be resultant from hypoestrogenic state from chemotherapy	Topical lubricants and vaginal estrogen can be used
Erectile dysfunction	May be resultant from damage to autonomic nerves and microvasculature from radiotherapy	Phosphodiesterase-5 inhibitors, optimization of other medical co-morbidities (HTN, DM), and urology referral for further evaluation can be considered
Body image distress	May result from cancer diagnosis, changes in bowel habits, or anatomic changes after surgery, such as an ostomy presence	Behavioral interventions, support groups, and counseling can be explored

*HTN* Hypertension, *DM* Diabetes Mellitus

### Psychosocial Distress

Recognition of psychosocial toxicity is an important cornerstone of rectal cancer survivorship. In a large, survey-based, observational study, the greatest long-term challenges identified by rectal cancer survivors include bowel/ostomy management (44%), negative psychosocial impact (37%), and long-term toxicity of treatment (21%) [81]. Psychosocial toxicity, evolving from physical impairments, emotional distress, and socioeconomic barriers, can manifest as pain, fear, anxiety, depression, isolation, and insomnia [82].

While QOL scores for rectal cancer survivors do typically improve over time, nearly one-third of patients report psychosocial distress at 5 years post-diagnosis [83]. Risk factors for psychosocial toxicity include pre-existing mood disorders, permanent ostomy, lower socioeconomic status, younger age, and physical inactivity [83–86].The presence of a permanent ostomy and impaired bowel function represents one of the largest sources and independent predictors of worse QOL and negative psychosocial well-being [27, 81, 85]. Rectal cancer survivors with ostomies are nearly 50% more likely to report depression than those without and report worse sexual function; physical barriers (see section above on the Sexual Dysfunction) and psychosocial barriers related to personal and inter-personal relationships around sexuality were identified on qualitative analyses [85, 87, 88].

In addition, patients with severe LARS are twice as likely to have high psychosocial distress and three times as likely to have low QOL compared to those with minor or no LARS [26, 86]. In rectal cancer survivors with significant symptoms from LARS, nearly 70% report significant job disruption and disability from frequent bowel movements, and 53% report financial stress [89]. Given the rising incidence of young-onset rectal cancer, providers should be attentive to financial toxicity and its psychosocial impact on younger patients who are less likely to have health insurance and may have larger financial commitments, such as dependent children.

The NCCN recommends all cancer survivors be screened for distress [82]. The NCCN Distress Thermometer is a one question tool, which asks patients to rate their level of distress in the past week on a scale of 0–10. Scores > 4 prompt the provider to elicit concerns about physical, emotional, social, functional, and spiritual well-being. Based on these responses, interventions ranging from reassurance and medication management to referrals for support groups, social work, physical therapy, mental health, and palliative care and symptom management services can be offered [82].

### Modifiable Risk Factors

Commonly identified risk factors for rectal cancer include excess body weight, physical inactivity, tobacco use, alcohol consumption, and high intake of red or processed meat [4, 90]. Table 3 provides a summary of lifestyle modifications that can lead to meaningful improvements in QOL and survival when integrated into survivorship care [91]. Rectal cancer survivors with higher composite lifestyle scores, which incorporates body mass index (BMI), diet, alcohol use, and smoking status, have a 46% reduction in all-cause mortality compared to those with lower composite lifestyle scores [92].

**Table 3:** Modifiable risk factors, recommendations, and their potential impact on cancer-specific outcomes and long-term toxicities

**Risk factor**	**Recommendations**	**Potential impact**
Diet modification	• Consume vegetables, fruits, and grains • Minimize intake of red meats, saturated fats, high sugar foods • High fiber diet if tolerated	• Reduction in all-cause mortality associated with healthy eating scores • Improved cardiovascular outcomes
Physical activity	• Aim for at least 150 min of physical activity per week	• Associated with reduced risk of recurrence and overall survival • Improved quality of life and reduced psychosocial distress • Reduced risk of osteoporosis with weight bearing exercises • Improved cardiovascular outcomes
Smoking	• Cessation of tobacco products	• Reduced risk of developing smoking related malignancies • Reduced all-cause mortality compared to former smokers • Improved cardiovascular outcomes • Reduced risk of osteoporosis • Improved erectile dysfunction severity
Alcohol intake	• Avoid or limit alcohol consumption to less than 3 standard drinks per day for men (2 for women)	• Reduced risk of developing alcohol related malignancies • Improved cardiovascular outcomes • Reduced risk of osteoporosis
Vitamin D/calcium supplementation	• Replete calcium and vitamin D to normal levels	• Improved progression free recurrence • Data on overall survival is mixed • Reduced risk of osteoporosis
Weight management	• Aim for healthy body mass index (BMI)	• Reduced risk of developing secondary obesity related malignancies • Improved control of other medical comorbidities, such as diabetes and cardiovascular disease

A diet rich in vegetables, fruits, and whole grains and low in red and processed meats, saturated fats, and high sugar foods is recommended by the American Cancer Society [93]. High fiber diets for patients without limiting gastrointestinal symptoms have also been associated with reduction in CRC specific and all-cause mortality [94]. Rectal cancer survivors with higher healthy eating scores compared to those with lower scores have a 40% reduction in all-cause mortality [92]. While many studies have shown that Western dietary patterns are associated with worse outcomes in colon cancer survivors, there is a lack of large RCT that demonstrate the impact of dietary changes on outcomes in rectal cancer survivors, specifically [91].

Physical activity ≥ 150 min per week is recommended by the American Cancer Society for all cancer survivors [93]. Increased physical activity in CRC survivors has been associated with reduced risk of recurrence, improved QOL, and improved overall survival [84, 95, 96]. In addition, there is growing evidence that elevated BMI in CRC survivors is associated with a 40% increased risk of secondary, obesity-associated cancers, such as post-menopausal breast, kidney, pancreas, esophageal, and endometrial cancer [97]. As such, weight loss to achieve a healthy body weight is recommended for all rectal cancer survivors.

Smoking cessation and limiting alcohol consumption are also both recommended lifestyle modifications for rectal cancer survivors. In CRC survivors who continue to smoke after diagnosis, all-cause mortality is nearly doubled compared to that of former smokers [98]. The impact of post-diagnosis alcohol consumption on mortality in CRC survivors is mixed; nonetheless, given the association between heavy alcohol use and osteoporosis, CVD, and new primary malignancies, survivors are recommended to avoid or limit alcohol consumption [93, 99].

Lastly, the data on vitamin D and calcium supplementation remain mixed. While high dairy and calcium intake has been associated with a lower mortality risk, this was not observed in patients taking vitamin D supplementation [100]. Low levels of vitamin D at diagnosis have been associated with inferior outcomes in colon cancer patients, though various RCTs have failed to show a clinically significant impact of supplementation on recurrence and survival [101]. However, a recent meta-analysis showed that vitamin D supplementation improved progression-free recurrence, though no benefit was seen in CRC survival [102]. Nonetheless, the American Cancer Society recommends adequate repletion of vitamin D and calcium levels in all patients [93], which we uphold as a low-cost intervention with minimal risk. We typically target repletion to 25(OH)D level of > 30 ng/dL.

## Conclusion

**Fig. 3:**
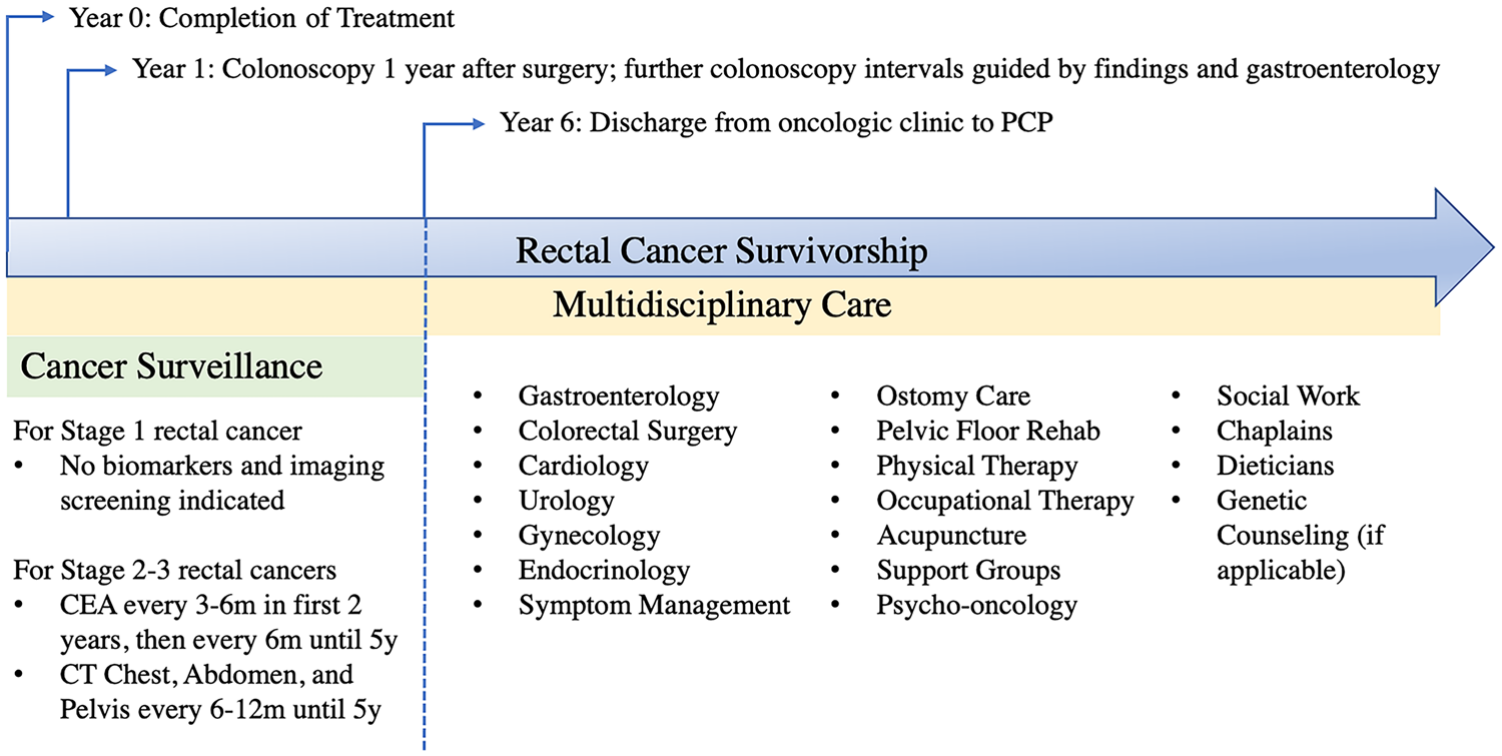
Rectal cancer survivorship extending beyond oncology clinic

Most patients will require significant support after undergoing surgery and/or chemotherapy and radiation therapy for rectal cancer. Long-term toxicity from cancer treatment can persist years after treatment. Early recognition and management of these adverse events during the survivorship phase of care can improve bowel function and other factors that contribute to QOL. We propose an integrated, multidisciplinary care approach (see Fig. 3) to aid rectal cancer patients and their providers in navigating through survivorship, which includes cancer surveillance recommendations based on the NCCN guidelines [5]. This approach integrates management of chronic toxicities and optimization of modifiable risk factors to reduce recurrence risk and comorbid conditions.
